# Biliary Sepsis Due to Recurrent Acute Calculus Cholecystitis (ACC) in a High Surgical-Risk Elderly Patient: An Unexpected Complication

**DOI:** 10.3390/pathogens11121423

**Published:** 2022-11-26

**Authors:** Giacomo Sermonesi, Alessia Rampini, Girolamo Convertini, Raffaele Bova, Nicola Zanini, Riccardo Bertelli, Carlo Vallicelli, Francesco Favi, Giacomo Stacchini, Enrico Faccani, Nicola Fabbri, Fausto Catena

**Affiliations:** 1 Department of Internal Medicine and Surgery (DIMEC), Alma Mater Studiorum, University of Bologna, S.Orsola-Malpighi Hospital, 40138 Bologna, Italy; 2 General and Emergency Surgery Department, Bufalini Hospital, Viale Ghirotti 286, 47521 Cesena, Italy

**Keywords:** biliary sepsis, gallbladder, bile duct, acute care, critical care, multidisciplinary, acute calculus cholecystitis, surgery, endoscopic ultrasound-guided gallbladder drainage (eus-gbd), gallstone ileus

## Abstract

Acute calculus cholecystitis (ACC) is increasing in frequency within an ageing population, in which biliary tract infection, including cholecystitis and cholangitis, is the second most common cause of sepsis, with higher morbidity and mortality rates. Patient’s critical conditions, such as septic shock or anaesthesiology contraindication, may be reasons to avoid laparoscopic cholecystectomy—the first-line treatment of ACC—preferring gallbladder drainage. It can aid in patient’s stabilization with also the benefit of identifying the causative organism to establish a targeted antibiotic therapy, especially in patients at high risk for antimicrobial resistance such as healthcare-associated infection. Nevertheless, a recent randomized clinical trial showed that laparoscopic cholecystectomy can reduce the rate of major complications compared with percutaneous catheter drainage in critically ill patients too. On the other hand, among the possibilities to control biliary sepsis in non-operative management of ACC, according to recent meta-analysis, endoscopic gallbladder drainage showed better clinical success rate, and it is gaining popularity because of the potential advantage of allowing gallstones clearance to reduce recurrences of ACC. However, complications that may arise, although rare, can worsen an already weak clinical condition, as happened to the high surgical-risk elderly patient taken into account in our case report.

## 1. Introduction

Acute calculus cholecystitis (ACC) is increasing in frequency within an ageing population [1], with 50–70% of cases occurring in elderly patients [2].

Biliary tract infection, including cholecystitis and cholangitis, is a cause of morbidity at any age, but it is particularly prevalent in elderly patients [3,4], in which it represents the second most common cause of sepsis after urinary tract infection [5]. The overall mortality rate of biliary infections is between 1% and 6%. However, when bacteremia is associated (10% of cholecystitis and 50% of acute cholangitis), mortality can reach 10% to 20% [6,7].

Early laparoscopic cholecystectomy (ELC) is recommended as first-line treatment of ACC [8]. This is evident in young as well as healthy patients but the management of ACC still remains controversial in high-surgical risk and frequently elderly patients.

Critical clinical conditions that make patients with ACC not suitable for surgery, such as septic shock or anaesthesiology contraindication, are reasons to avoid ELC and to prefer performing gallbladder drainage (GBD), as it converts a septic patient with ACC into a non-septic patient [8].

Gallbladder drainage decompresses the infected bile or pus in the gallbladder and it can result in reduced inflammation and in improvement of the clinical conditions. Furthermore, identifying the causative organism (positive rates of either bile or gallbladder cultures range from 29 to 54% for acute cholecystitis) is an essential step in the management of ACC, especially in patients at high risk for antimicrobial resistance such as healthcare-associated infections [9]. The main antimicrobial resistance is due to extended spectrum beta-lactamase (ESBL) producing Enterobacteriaceae. It is found frequently in community acquired infections in patients with comorbidities requiring frequent exposure to antibiotic treatments [10].

Nevertheless, a systematic review comparing percutaneous gallbladder drainage (PC-GBD) and cholecystectomy in critically ill patients reported highly mortality rate, lenght of hospital stay and number for readmission in the PC-GBD group than in cholecystectomy group [11].

Furthermore, a recent randomised clinical trial (the CHOCOLATE trial) showed that ELC reduced the rate of major complications also in critically ill patients (APACHE score 7–14) compared to PC-GBD [12].

On the other hand, among the GBD techniques to control biliary sepsis, endoscopic ultrasound-guided gallbladder drainage (EUS-GBD) is gaining popularity in the non-operative management (NOM) of high surgical-risk patients with ACC. A recent meta-analysis [12] showed that it has better clinical success rate than Endoscopic Transpapillary Gallbladder Drainage (ET-GBD) and PC-GBD, with comparable pooled rates of adverse event (e.g., post-procedural pancreatitis more frequent with ET-GBD, bleeding and perforation more frequent with EUS-GBD, stent migration more frequent with PC-GBD). Moreover, the rate of disease recurrence was comparable between ET-GBD (4.6%) and EUS-GBD (4.2%) and significantly inferior to PC-GBD (10.8%).

However, these findings are based on data from high-volume centres where only a small number of skilled physicians perform this procedure and the most appropriate post-procedural management of the patient that receives EUS-GBD for ACC is still unclear. Once cholecystitis is resolved, should the stent be removed? Is there an optimal duration of stenting? How to prevent ACC recurrence? Persistence of Self-expandable metallic stent (SEMS) or Lumen-apposing metal stents (LAMS) even up to three years with few adverse effects has been observed in some studies, suggesting that permanent stenting could be an option at least in patients with minimal life expectancies or high-risk comorbidities [13,14,15]. On the other side Kamana et al. [16] showed that the replacement of SEMS, after approximately one month and improvement of symptoms, by a double-pigtail plastic stent, was effective in reducing recurrence of acute cholecystitis. In fact, this complication seems to occur secondarily to stent dislodgement or occlusion from gallstones, food bolus, clots, or tissue overgrowth over time. A few authors recommend prophylactic placement of pigtail stents through the LAMS at the time of initial procedure to prevent this complication [17,18,19,20].

A further possibility is represented by cholecystoscopies with the aim of complete stone clearance as well as to replace the LAMS with double pigtail plastic stent to prevent future attacks [21]. Larger stones cannot be extracted directly because of the limited space in the saddle section of the LAMS, so endoscopic laser lithotripsy and lithotomy (ELLL) may be a good method to remove gallstones through LAMSs, especially for giant gallstones, also in order to reduce the rates of recurrences [22,23].

However, complications that may arise, although rare, can worsen an already weak clinical condition.

## 2. Case Report

We present the case of a severely cardiopathic 75-year-old admitted to the emergency department because of the development of a deep upper right abdominal pain and fever.

The patient reported having had a couple of episodes in the last year of similar but much milder symptoms treated with antibiotic therapy on the recommendation of the general practitioner.

**Figure 1:**
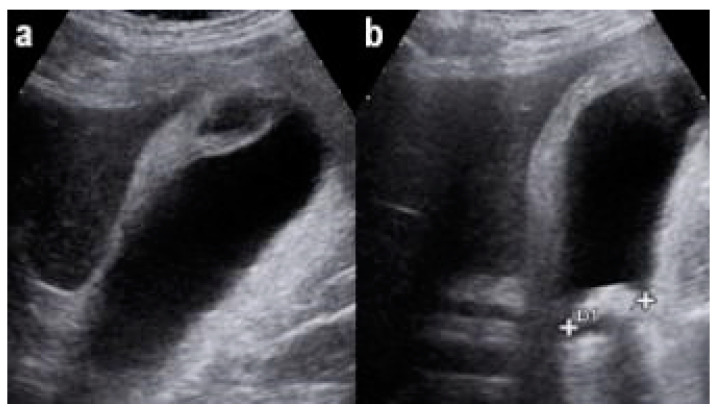
ACC with focal wall necrosis (**a**) and a big gallstone (diameter 30 mm) (**b**).

The patient met qSOFA criteria: score was 2 (Respiratory rate 22 and systolic blood pressure 95 mmHg) and an abdominal ultrasound exam (Figure 1) suggested an ACC with focal wall necrosis and a big gallstone (diameter 30 mm).

The patient met sepsis criteria (T 38.5 °C, Respiratory rate 24, WBC 16,000/mm^3^ and presence of biliary source infection). Despite the absence of hemodynamic instability, with a satisfactory increase in blood systolic pressure after adequate intravenous hydration, the decision of avoiding early laparoscopic cholecystectomy (ELC) was taken because of anaesthesiology contraindication due to Heart Failure with reduced Ejection Fraction (LVEF 25%).

Non-operative treatment was chosen with an immediate start of empiric antibiotic therapy, observing an initial clinical laboratory improvement. Forty-eight hours after hospitalization, following a further episode of shivering fever and a new increase in inflammation and cholestasis indices, the patient underwent endoscopic ultrasonography (EUS) that showed the already known acute cholecystitis with 5 mm gallstone in the common biliary duct. Sphincterotomy and extraction of the gallstone with a Fogarty balloon were conducted. After excluding the interposition of vessels and evaluation of the distance between the duodenal wall and the lumen of the gallbladder (13 mm), EUS-GBD with LAMS (Hot-AXIOS 15 × 15 mm) was successfully performed with spillage of bile and abundant pus. The patient experienced relief of symptoms with progressive decrease in inflammatory index and he was discharged four days after the procedure.

**Figure 2:**
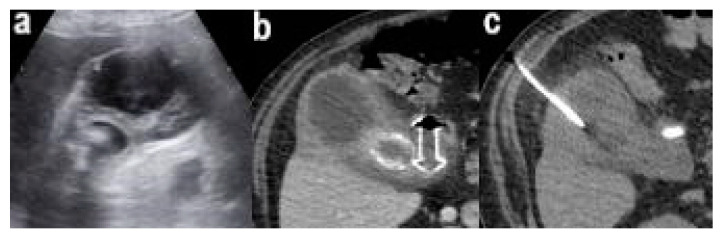
Abdominal US showed an ACC with a 50 mm abscess of the wall and the known big gallstone 37 × 30 mm (**a**), CT scan show Hot-Axios positioned between the gallbladder and duodenum (**b**), CT scan-guided PC-GBD (**c**).

After 42 days of well being the man had another recurrence of ACC and once readmitted to the emergency department, he underwent an abdominal ultrasound exam and an abdominal CT scanning that suggested an acute cholecystitis with a 50 mm abscess of the wall and the known big gallstone 37 × 30 mm (Figure 2). Endovenous empiric antibiotic treatment was started and he underwent a PC-GBD that allowed the evacuation of gallbladder wall abscess.

A culture swab was performed from the exudate coming out from the percutaneous drainage, which was positive for multi-drug resistant Enterococcus, therefore the patient started a targeted antibiotic therapy with Vancomycin. The drainage remained correctly positioned for five days but then the patient autoremoved it during an episode of acute delirium. No complications occurred and the patient was discharged after ten days from admission.

One week later the patient was readmitted to the hospital for SARS CoV-2 Pneumonia with respiratory failure. The patient overcame the disease and after a month of hospitalization he was discharged.

**Figure 3:**
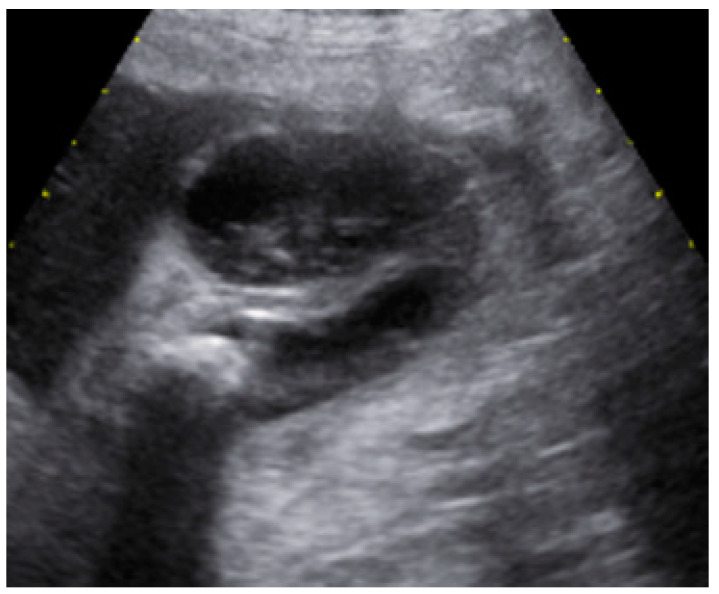
Abdominal US exam showed pericholecystic fluid collection (60 mm of diameter) in ACC.

After a week since his discharge, the patient entered again the emergency room for the third severe recurrence of ACC in the last three months. An abdominal US exam showed a pericholecystic fluid collection (60 mm of diameter) in ACC (Figure 3).

The patient refused PC-GBD as he had not previously tolerated it, but he restarted a targeted antibiotic therapy based on culture swab exudate from previous PC GBD. The patient benefited from antibiotic therapy and in order to prevent ACC recurrence the patient underwent endoscopy with the aim of gallstone clearance. ERCP with regular opacification of the biliary tract and cystic duct was conducted. The Hot-AXIOS prosthesis was then removed and Electrohydraulic lithotripsy with Autolith was afterwards performed, obtaining partial fragmentation of the voluminous endocholecystis stone and removal of the fragments. Subsequently, 7 FR double pig-tail prosthesis were placed. The patient was discharged following 4 days of good general conditions.

**Figure 4:**
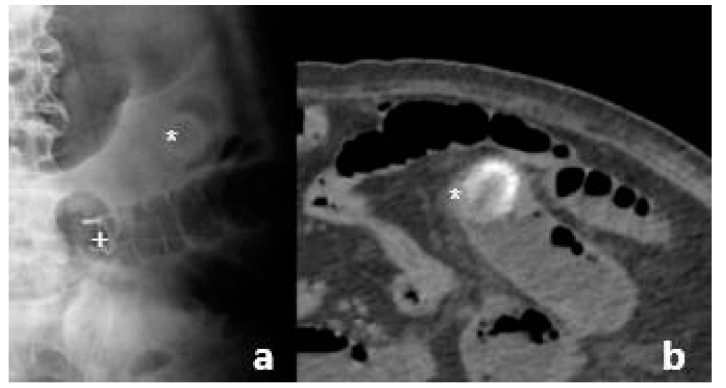
Abdominal X-ray (**a**) and abdominal CT scan (**b**) showed pig-tail prothesis (+) and gallstone (*) in small intestine.

After 14 days of well-being, the patient went once again to the emergency room for pain and abdominal distension and constipation. An abdomen X-ray showed the double pig- tail prothesis dislocation in a jejunal tract and a left hypochondrium roundish radiopaque image referable to endocolecystic calculus migrated to the intestine. An in-depth diagnostic abdominal CT scan confirmed biliary ileus and double pig-tail prothesis ileus displaced in jejunum (Figure 4).

The surgical option was discussed with the patient and his family members, informing of the high risk of perioperative morbidity and mortality (POSSUM mortality 26.5%, morbidity 80.7%). The patient agreed to surgery.

**Figure 5:**
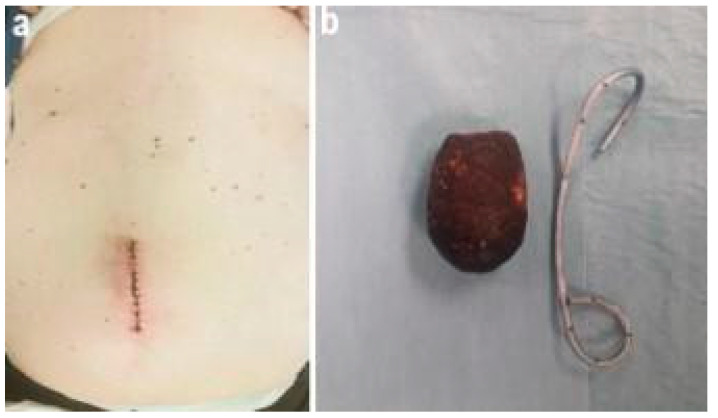
Supraumbilical laparotomy (**a**), gallstone and double pigtail (**b**).

The decision was made for a laparotomic approach considering the severely altered systolic function and the important abdominal distension. The patient underwent supraumbilical laparotomy, transverse jejunal enterotomy, gallstone and double pigtail extraction and longitudinal enterotomy suture according to Heineke-Mikulicz (Figure 5).

At the end of the procedure the patient was admitted to IC-Unit. After 24 h of intensive cardio-respiratory monitoring, the patient returned to the Surgical Ward. The patient had an unexpectedly regular postoperative hospitalization with no significant medical-surgical events, and he was discharged on the 5th postoperative day.

## 3. Discussion

We presented the case of a severely-cardiopathic elderly man who developed a biliary sepsis due to severe ACC. It was decided to avoid early laparoscopic cholecystectomy (ELC) because of anaesthesiology contraindication due to Heart Failure with 25% Left Ventricular Ejection Fraction. Non-operative management with empiric anti-antibiotic therapy was chosen with initial clinical laboratory improvement observed. Forty-eight hours after hospitalization, following a further episode of shivering fever and a new increase in inflammation and cholestasis indices, the patient underwent ERCP with common biliary duct gallstone extraction and endoscopic ultrasound guided gallbladder drainage (EUS-GBD) with lumen apposing metal stent (LAMS) which was clinical successful. EUS-GBD has proved effective in the management of biliary sepsis during the first episode of severe ACC, but it inevitably leads to the formation of a duodenal gallbladder fistula. A PC-GBD was requested to treat the second ACC recurrence due to a gallbladder wall abscess formation, together with a targeted antibiotic therapy with Vancomycin, based on a multi-drug resistant Enterococcus isolated from a drainage colture. Approximately one month later, in the hypothesis that the stent occlusion or the persistence of the large gallstone could be the cause of the third ACC recurrence, LAMS was replaced with double pig-tail prosthesis and endoscopic electrohydraulic lithotripsy was performed, obtaining only partial fragmentation and removal of gallstone. The patient subsequently developed a gallstone ileus due to the migration of the stone and the double pig-tail prosthesis, requiring urgent surgery.

Although our case is singular and the patient survived the adverse event with an unexpectedly regular postoperative course, it is necessary to consider that EUS GBD, by the creation of a iatrogenic fistula between the gallbladder and the gastrointestinal tract, may lead to the development of a gallstone ileus in a high surgical-risk patient, even more after endoscopic revisions procedures which might become necessary in case of recurrences of acute cholecystitis.

In a recent meta analysis on high surgical-risk patients with ACC treated with gallbladder drainage, Mohan BP et al. [13] showed that EUS-GBD had better technical and clinical success rates than Endoscopic Transpapillary Gallbladder Drainage (ET-GBD) and PC-GBD, with comparable pooled rates of adverse event. However, the pooled all-cause mortality rate with EUS-GBD was significantly greater (26%) when compared with ET-GBD (16%) or PC-GBD (11.2%). Even in a recent systematic review on 189 patients who underwent EUS-GBD with LAMS, Jain D et al. [24] collected possible related complications and therapeutic measures taken in each situation. Complications, although rare, were associated with the risk of worsening the clinical situation in already sick patients. Although some authors [16,21,22,23] support that cholecystoscopies for gallstone clearance, especially for giant gallstone, and replacement of the LAMS with double pigtail plastic stent, could be effective to prevent future attacks, new evidences are needed on feasibility and safety of these procedures.

In a recent randomized clinical trial, the CHOCOLATE trial [12], 142 high risk patients with acute calculous cholecystitis were randomly allocated to laparoscopic cholecystectomy (*n* = 66) and percutaneous catheter drainage (*n* = 68). ELC, compared with PC- GBD, had low rate of major complication, that occurred in eight of 66 patients (12%) versus 44 of 68 patients (65%) assigned to PC- GBD.

The CHOCOLATE trial, reasserted the importance of ELC in high risk patients with ACC too, defined as an acute physiological assessment and chronic health evaluation II (APACHE II) score of 7 or more.

It becomes necessary to further analyze the comorbidities and the conditions that increase the perioperative risks, especially in elderly patients.

In a recent retrospective study on emergent cholecistectomy for ACC, Serban et al. [25], found that surgical complication had higher correlation with systemic comorbidities: diabetes (r = 0.813) and chronic bronchopneumopathy (r = 0.502) and CCI (r = 0.381, but with no significant increase in discrimination power). Among the local factors, the severity of inflammation and the presence of gangrenous cholecystitis had the most significant predictive power (r = 0.288), followed by fibrinogen (r = 0.348), and TG13/TG18 severity forms (r = 0.218).

## 4. Conclusions

The management of biliary sepsis due to ACC in high-surgical risk and elderly patients is still controversial and it represents a challenge for clinicians, requiring therefore a multidisciplinary approach.

Non-operative management (NOM) including GBD procedures, may help in patient’s stabilization when critical conditions avoid early laparoscopic cholecystectomy (ELC), with the benefit of identifying the causative organism in order to establish a targeted antibiotic therapy. EUS-GBD is gaining popularity in NOM because of a high clinical success rate and the potential advantage of allowing endoscopic revision with gallstones clearance, in case of ACC recurrences. Nevertheless, these findings are based on data from high-volume centers and more data are needed on new endoscopic procedures, because complications that may arise, although rare, could be associated with the risk of worsening the clinical situation in already critical patients who are poor surgical candidates, as in this case report.

In critical patient with biliary sepsis due to ACC, it is essential to seek an estimate of the real ratio between benefits and risks that could result from surgery, as ELC remains the only therapeutic choice that can allow a forward and effective removal of the source of infection, and according to recent studies even high-risk patients can benefit from it.

Additional trials are needed to further identify conditions and comorbidities that increase the perioperative risks especially in elderly patients, in order to address clinical decisions towards a tailored therapy.

## Data Availability

Not applicable.
